# Real World and Public Health Perspectives of Intraoperative Radiotherapy in Early-Stage Breast Cancer: A Multidisciplinary Analysis Beyond the Statistical Facts

**DOI:** 10.7759/cureus.36432

**Published:** 2023-03-20

**Authors:** Srinivasan Vijayakumar, Mary R Nittala, Vedanth Buddala, Paul Mobit, William N Duggar, Claus Chunli Yang, Seth T Lirette, Eswar Mundra, Hiba Z Ahmed, Scott M Berry, Barbara S Craft, William C Woods, Jeremy Otts, Asal Rahimi, Thomas Dobbs

**Affiliations:** 1 Radiation Oncology, University of Mississippi Medical Center, Jackson, USA; 2 Data Science, University of Mississippi, Jackson, USA; 3 Surgery, University of Mississippi Medical Center, Jackson, USA; 4 Medicine, University of Mississippi Medical Center, Jackson, USA; 5 Radiation Oncology, University of Texas Southwestern Medical Center, Dallas, USA; 6 Population Health, University of Mississippi Medical Center, Jackson, USA

**Keywords:** accelerated partial breast irradiation, breast cancer care, breast conservation therapy, early-stage breast cancer, intraoperative radiotherapy

## Abstract

Breast conservation therapy (BCT) (usually a lumpectomy plus radiotherapy (RT)) has become a standard alternative to radical mastectomy in early-stage breast cancers with equal, if not higher, survival rates. The established standard of the RT component of the BCT had been about six weeks of Monday through Friday external beam RT to the whole breast (WBRT). Recent clinical trials have shown that partial breast radiation therapy (PBRT) to the region surrounding the lumpectomy cavity with shorter courses can result in equal local control, survival, and slightly improved cosmetic outcomes. Intraoperative RT (IORT) wherein RT is administered at the time of operation for BCT to the lumpectomy cavity as a single-fraction RT is also considered PBRT. The advantage of IORT is that weeks of RT are avoided. However, the role of IORT as part of BCT has been controversial. The extreme views go from “I will not recommend to anyone” to “I can recommend to all early-stage favorable patients.” These divergent views are due to difficulty in interpreting the clinical trial results. There are two modalities of delivering IORT, namely, the use of low-energy 50 kV beams or electron beams. There are several retrospective, prospective, and two randomized clinical trials comparing IORT versus WBRT. Yet, the opinions are divided. In this paper, we try to bring clarity and consensus from a highly broad-based multidisciplinary team approach. The multidisciplinary team included breast surgeons, radiation oncologists, medical physicists, biostatisticians, public health experts, nurse practitioners, and medical oncologists. We show that there is a need to more carefully interpret and differentiate the data based on electron versus low-dose X-ray modalities; the randomized study results have to be extremely carefully *dissected* from biostatistical points of view; the importance of the involvement of patients and families in the decision making in a very transparent and informed manner needs to be emphasized; and the compromise some women may be willing to accept between 2-4% potential increase in local recurrence (as interpreted by some of the investigators in IORT randomized studies) versus mastectomy. We conclude that, ultimately, the choice should be that of women with detailed facts of the pros and cons of all options being presented to them from the angle of patient/family-focused care. Although the guidelines of various professional societies can be helpful, they are only guidelines. The participation of women in IORT clinical trials is still needed, and as genome-based and *omics*-based fine-tuning of prognostic fingerprints evolve, the current guidelines need to be revisited. Finally, the use of IORT can help rural, socioeconomically, and infrastructure-deprived populations and geographic regions as the convenience of single-fraction RT and the possibility of breast preservation are likely to encourage more women to choose BCT than mastectomy. This option can also likely lead to more women choosing to get screened for breast cancer, thus enabling the diagnosis of breast cancer at an earlier stage and improving the survival outcomes.

## Introduction and background

Intraoperative radiotherapy (IORT) for early-stage breast cancer (EBC) as part of breast conservation therapy (BCT) is no doubt a very controversial subject in breast cancer care today [1]. Opinions rendered sometimes take extreme *polar *points. Thus, taking a nuanced approach to healthcare decision-making can be lost in the heat of the debate. Making clinical decisions is a *scientific art*. In this *scientific art* of making clinical decisions for our patients, one often has to weigh (a) the scientific evidence; (b) the statistical versus the actual, practical real-world clinical impact of studies; (c) the individual patients and his/her family’s circumstances and choices; (d) geography and infrastructure options available for the patient and the facility; and, finally, (e) cost-benefit and risk-benefit considerations. Furthermore, the importance of patient and family-focused care and fully involving patients and families in decision-making in the process is gaining momentum [2] in this age of expanding information technology use in health care [3], extended use of telemedicine, and considering public health perspectives including methods to overcome disparities in healthcare delivery to rural and socioeconomically deprived populations (RSEDPs).

In this multidisciplinary review and perspective, an attempt is made to apply the above concepts to arrive at a consensus that is patient and family-centered, pragmatic, and practical with an aim to gain maximum health benefits not only in terms of survival but also in the quality of life (QOL) that incorporates organ and function preservation as well as increases patient satisfaction in the physical and mental health outcomes/domains. A multidisciplinary team of radiation oncologists, breast surgeons, medical physicists, medical oncologists, nurse practitioners, biostatisticians, and public health experts have worked together to carefully investigate the multiple dimensions in presenting the data and scientific results. This is done in a way that can help healthcare providers, patients, families, and healthcare facilities to make decisions in a comprehensive and nuanced way that is practical, ethical, and leads to the least harm and maximum benefit from the perspective of women with EBC, and not just from the dry statistical point of view. The potential public health benefits in terms of increasing breast cancer screening compliance among RSEDPs by giving women the confidence that the diagnosis of breast cancer is not a death sentence and would not automatically and invariably lead to mastectomy and loss of self-esteem by providing them with choices that are not *cookie-cutter* *straight-jacket* approaches of a *single-size-fits-all* decision-making process.

In 2022, an estimated 300,000 breast cancers were diagnosed, including invasive and non-invasive cancers, in the United States, and 43,780 patients died from the disease [4]. Due to the widespread use of mammography screening, two-thirds of breast cancers are now diagnosed in the early stages [5]. Currently, the standard of care for EBC is breast-conserving surgery (BCS), followed by adjuvant whole-breast external beam radiotherapy (EBRT) or mastectomy alone [6,7]. The adjuvant EBRT to the whole breast, including the cavity, is administered over five weeks, followed by a lumpectomy cavity boost to an additional one-week course of radiation therapy (RT) depending upon the risk criteria/factors to suppress local recurrence [8].

Due to the need to make daily clinic visits for RT for six weeks, the socioeconomic burden/financial toxicity [9] increases for women and their families, and women’s QOL declines. In many countries, patients cannot complete weeks of EBRT, especially those living far from RT centers [10]. Furthermore, depending on the volume of breast tissue being irradiated, which depends on many factors (including breast size), there will be cosmetic consequences on the breast such as pigmentation, induration, skin atrophy, breast size changes compared to the opposite breast, and potential RT-induced toxicity to the lungs and heart [11].

Because a series of observational studies have shown that most local recurrences occur at or near the original site of breast cancer, a paradigm shift has occurred in breast cancer RT practices for early, favorable prognostic patients. This includes a trend of global practice and patients’ preference favoring hypofractionated (to be explained later) RT schedules. Partial breast radiation therapy (PBRT) is a type of RT using de-escalation to reduce the number of days of visits to RT centers and decrease the volume of RT for patients with a low risk of local recurrence. The concept of PBRT is based on the evidence that most local, in-breast recurrences occur in the breast tissue adjacent to the lumpectomy cavity [12].

IORT is a form of PBRT that can deliver a large, single dose of radiation to the resection cavity and tissues adjacent to the cavity at the time of operation to the immediate tumor bed (less than 3-4 mm). The advantages are precise RT, protecting healthy tissues, avoiding treatment delay, and preventing weeks of visits to RT centers.

This paper aims to bring nuances into the decision-making process in favor or against using IORT in EBC, including incorporating the input of patients/families with fully informed consent. In addition, we would like to clarify the conversation by including stakeholders beyond breast surgeons and radiation oncologists to decrease the bias in our opinions. Finally, we will point out potential areas of further research, especially in the public health arena on the potential benefits of educating women about not having to undergo a mastectomy with early diagnosis, thus improving mammographic screening rates [13].

Our hypotheses are (a) by judicially selecting IORT with fully informed consent, we will make more women come forward to get BCS + (IO)RT. This will give equal survival outcomes and perhaps slightly lower in-breast control than whole breast radiotherapy (WBRT) after BCS. (b) Our multidisciplinary approach is likely to remove many ongoing controversies due to the misunderstanding of statistical interpretations, dosimetric differences between low-energy 50 kV X-rays versus electrons, patient selection criteria in clinical trials, and the confusion caused by multiple guidelines from different societies. (c) When women know that mastectomy is not the only option, they will come forward more willingly for mammographic screenings. This will lead to more early cancer diagnoses and will have a beneficial public health effect.

Some of the discussions and explanations may sound too basic for radiation oncologists and other breast cancer oncologists, which is true. However, we would like our audience to be more diverse for this communication, including internists, family practitioners, breast geneticists, medical physics colleagues, breast cancer nurse practitioners and physician assistants, social workers, nurses taking care of breast cancer patients, and especially public and population health professionals, cancer center administrators, patient educators, and cancer care navigators.

## Review

High rates of mastectomy in rural and socioeconomically deprived populations

Data indicate that the rates of mastectomy are higher for equivalent stages of breast cancer in rural areas compared to urban regions. Through a 2006 study using a SEER database, a retrospective analysis showed an increased mastectomy rate in rural populations when compared to urban populations [14]. Cancer stages I-III were analyzed, and different variables such as age, income, education, and the number of radiation facilities were considered. Some significant factors that affected this conclusion were employment, education, and the number of radiation oncologists in the areas examined. All significant variables were directly correlated with lower levels in rural areas creating a disparity [15]. Viewing things from a larger perspective, when examining breast cancer worldwide, a significant distinction was shown between urban and rural populations. Through the analysis of different surgical treatments in multiple countries, of 16 studies from the United States, three from Australia, two from Canada, one from China, and one from Japan, it was seen that urban areas were significantly more likely to receive BCS or other more selective surgery compared to mastectomy [14]. It was also seen that rural areas with BCS had low rates of follow-up, adjuvant RT. The influence of traveling and cost considerations have also created a distinction between rates of mastectomy in rural and urban areas [16].

One needs to understand the importance of decision-making for rural areas affected by cancer. Any chance for improvement in financial stability or convenience in obtaining cancer treatment can easily impact decisions for these populations. Because of these factors, rural patients are significantly more likely to choose mastectomies currently because of the current *so-called standard* practice of daily RT for five to six weeks. This is a great inconvenience for patients and families. This inconvenience leading to avoiding RT is illustrated by comparing travel times and completion of five to six weeks of RT [10]. Patients living a distance of greater than 10 miles were less likely to complete RT [10]. Fourteen individual studies also supported that women living further away from RT facilities and treatment centers are less likely to receive BCS and more likely to receive mastectomy [16]. Rural residents have difficulties accessing transportation. This study further shows that distances can cause rural (and even urban) residents to choose mastectomy over breast preservation treatments to avoid the inconvenience and life disruptions induced by weeks of RT [16].

Through analyzing the density of radiation oncologists, six studies in the United States showed significant differences between urban and rural areas, an increasingly *divided gap* in the choice of treatments because it is more difficult for rural patients to access essential radiation facilities. Although the percentage of US radiation facilities has increased in the past 15 years, urban areas already dense with these facilities have been disproportionately favored [17]. These observations are supported by studies showing that BCS is frequently common among women living in areas with more health facilities. Mammograms and breast screening are also difficult with rural populations because of the lack of available resources, which causes higher mastectomy rates [16]. As cancer progresses more rapidly, a lack of breast cancer screening facilities causes mastectomy to be an efficient option for rural patients because of the advanced stage and being ineligible for BCS. For similar reasons, rural patients with lower socioeconomic status are less likely to be willing to choose a prolonged RT course over mastectomy, even when eligible for BCT.

A brief review of equal (or improved survival) results of breast conservation therapies in early-stage and favorable prognostic breast cancers among women

According to the National Comprehensive Cancer Network guidelines, the current standard of care treatment for early-stage, invasive breast cancer (T1-T3, ≥N0, M0) includes either BCS followed by RT or mastectomy ± RT [18]. For T1-T3, ≥N0, M0 patients who choose to undergo mastectomy, chest wall RT ± regional nodal irradiation is indicated in patients with positive nodes and those with multiple high-risk pathological components for recurrence. High-risk factors for recurrence include central/medial tumors or tumors ≥2 cm with <10 axillary nodes removed with at least one of the following factors: grade 3, estrogen receptor (ER)-negative, or lymphovascular invasion. For very favorable cases of EBC (cT1-3, N0, M0 without any high-risk factors for recurrence), treatment with total mastectomy with surgical axillary staging alone is sufficient.

Several studies have established that for very favorable cases (cT1-T3, ≥N0, M0), BCS (BCS followed by RT) has equivalent long-term results compared to mastectomy alone [19-22].

**Table 1:** Randomized and pooled data comparison of mastectomy versus breast conservation therapy. NSABP: The National Surgical Adjuvant Breast and Bowel Project; EBCTCG: The Early Breast Cancer Trialists’ Collaborative Group; BCS: breast-conserving surgery; RT: radiation therapy; %: percentage

Study	Center/Country	Inclusion years	Number of patients	Inclusion criteria	Follow-up	Treatment arms	Outcomes
NSABP [19]	United States	1976–1984	Total: 1,851; total mastectomy: 589; lumpectomy alone: 634; lumpectomy plus irradiation: 628	Size: 4 cm or less, stage I or II	20 years	Total mastectomy, lumpectomy, lumpectomy plus irradiation	Local recurrence = lumpectomy alone: 39.2%; lumpectomy plus irradiation: 14.3%; p < 0.001
Milan study [20]	Italy	1973–1980	Total: 701; mastectomy: 349; BCS followed by RT: 352	Size: >2 cm, stage T1 N0	20 years	Radical mastectomy, BCS with RT	Local recurrence = radical mastectomy 2.3%; BCS with RT: 8.8%; p < 0.001
EBCTCG [21]	United Kingdom	1985–2000	Total: 42,100; RT vs. no RT: 23,500; more vs. less surgery: 9,300; more surgery vs. RT: 9,300	N/A	15 years	RT vs. no RT, more vs. less surgery, more surgery vs. RT	Local recurrence = BCS + RT: 7%; BCS alone: 26%; RT reduced five-year local recurrence by 19%
Swedish National Study [22]	Sweden	2008–2017	Total: 48,986; BCS + RT: 29,367; mastectomy alone: 12,413; mastectomy with RT: 7,206	T1-2 N0-2	6 years	BCS + RT, mastectomy alone, mastectomy with RT	Overall survival = BCS + RT: 95.1%; mastectomy alone: 84.5%; mastectomy with RT: 86%

Once the role of BCT and RT was established in the treatment of EBC to be equivalent (if not superior in terms of long-term survival, as shown in the above two large cohort studies [21,22]), goals shifted toward decreasing the time interval over which RT is given with hypofractionated doses and fractionation schemas.

Hypofractionated radiation therapy after breast-conservation surgery

In 1993, Wheelan et al. started accruing patients diagnosed with EBC (T1-2, N0) for a study that compared the conventional fractionation regimen of 50 Gy in 25 fractions to a moderate hypofractionated regimen of 42.5 Gy in 16 fractions. All patients received BCS with negative margins and axillary dissection with no positive nodes and were randomized to one of the fractionation schemas [23]. In total, 622 women were randomized to the 42.5 Gy in 16 fractions arm, and 612 patients were randomized to the control arm, which was 50 Gy in 25 fractions. The five-year recurrence-free survival for patients in the hypofractionated and standard fractionation arms was 97.2% and 96.8%, respectively. However, at 10 years, the local failure rate for the hypofractionated arm was 6.2% and 6.7% for the control arm. There was also no statistically significant difference in cosmesis between patients in each arm.

The START (UK Standardization of Breast Radiotherapy) Trialists’ Group conducted similar trials. START A compared conventional fractionation (50 Gy in 25 fx) to 39 Gy in 13 fractions and 41.6 Gy in 13 fractions [24]. START B compared standard fractionation (50 Gy in 25 fx) to 40 Gy in 15 fractions. The 10-year locoregional failure rate across all arms of both studies ranged from 4% to 6% with no statistically significant differences. Cosmetic outcomes were either the same or improved in the hypofractionated arms. Specifically, the rate of breast edema was statistically significantly decreased by 46%, telangiectasias by 57%, and breast induration by 24% (hazard ratio (HR) = 0.54, 0.43, and 0.76, respectively).

The Danish Breast Cancer Group (DBCG) conducted a large, non-inferiority, randomized, controlled trial comparing conventional fractionation of 50 Gy in 25 fractions to 40 Gy in 15 fractions in node-negative breast cancer/ductal carcinoma in situ (DCIS) patients receiving BCS [25]. The nine-year risk of locoregional recurrence was 3.3% (95% confidence interval (CI) = 2.0% to 5.0%) in the 50-Gy group and 3.0% (95% CI = 1.9% to 4.5%) in the 40-Gy group (risk difference = -0.3%; 95% CI = -2.3% to 1.7%). The nine-year overall survival was 93.4% (95% CI = 91.1% to 95.1%) in the 50-Gy group and 93.4% (95% CI = 91.0% to 95.2%) in the 40-Gy group. Again, cosmetic outcomes were more favorable in the hypofractionated regimen. With the publication of this DBCG-HYPO trial, 40 Gy in 15 fractions became an accepted regimen for post-BCS surgery as the standard of care.

The UK FAST-Forward trial took moderate hypofractionation even further [26]. FAST-Forward was a multicenter, phase 3, randomized, non-inferiority trial conducted across 97 sites across the United Kingdom. Women with EBC, which was defined as T1-T3, N0-1, who decided to undergo BCS were eligible for the trial. The trial compared two ultra-hypofractionated regimens, 26 Gy in five fractions and 27 Gy in five fractions, to the newly added standard of care, 40 Gy in 15 fractions. A total of 1,361 women were assigned to the 40-Gy schedule, 1,367 to the 27-Gy schedule, and 1,368 to the 26-Gy schedule. Although we only have five-year data for the results published from this trial as opposed to the nine or 10-year data from the trials mentioned above, the outcomes are promising. There was no difference in ipsilateral breast cancer recurrence between any of the fractionation schemas (HRs of 0.86 (95% CI = 0.51 to 1.44) for 27 Gy in five fractions and 0.67 (95% CI = 0.38 to 1.16) for 26 Gy in five fractions when compared to the control arm of 40 Gy in 15 fractions). Although there was no difference in the ipsilateral breast cancer recurrence rates, cosmesis outcomes varied between the three arms. Women who received 27 Gy had worse late adverse radiation-induced side effects with an odds ratio (OR) of 1.55 (p < 0.0001). Late toxicities included breast shrinkage, distortion, induration, edema, and telangiectasias. There was no statistically significant difference in late toxicities between the 26-Gy and 40-Gy arms. We await the publication of the 10-year data.

The above data led many professional societies in the United States and Europe to develop guidelines and consensus statements. For example, the American Society of Radiation Oncology (ASTRO) issued guidelines for PBRT based on age, tumor size, margin, and other risk factors in 2009 [27]. These guidelines enunciated three subcategories, namely, those suitable, cautionary, and unsuitable for PBRT [28]. The results of many subsequent studies following these guidelines and analyzing the results led to IORT also being accepted as a type of PBRT. PBRT can be given in 5-15 fractions as EBRT or single IORT treatment. The words *partial breast* in PBRT means only a small volume of the breast is irradiated. As is discussed elsewhere in this paper, treating small volumes with PBRT leads to fewer complications and better cosmetic results. In addition, IORT treats even less volume of normal tissues than external beam-based PBRT. In the following sections, the data from IORT in terms of outcomes as well as the nuances of dosimetric aspects will be discussed.

Role of intraoperative radiation therapy in early-stage breast cancer as part of breast conservation therapy: results of a single institution and multi-institutional randomized studies

Table 2 shows the outcomes of non-randomized (retrospective and prospective) studies using low-energy kV photons in IORT. Some of the studies were started as early as 2000 with long years of follow-up data and very mature results, whereas others were started as late as 2022. The patient numbers also vary from fewer than 100 to over 1,000. However, one can conclude a very unison finding of ipsilateral breast tumor recurrence (IBTR) being around 2% with a few outliers of about 4% and 6% [29-36].

**Table 2:** Outcomes from single/multi-institutional prospective and retrospective studies on intraoperative radiotherapy using kV photons in early-stage breast cancer after breast-conserving surgery (lumpectomy/quadrantectomy). Single: single institution; Multi: multi-institutional; IBTR: ipsilateral breast tumor recurrence; %: percentage

Author/year	Center/Country	Inclusion period	Single/multi-institution	Number of patients	Technique	Median follow-up years	Five-year IBTR rate
Silverstein et al., 2018 [29]	Hoag Memorial Hospital, Presbyterian, US	2010–2017	Single	984	Photon (50 kV)	3	3.9%
Tejera Hernández et al., 2020 [30]	Hospital Universitario, Spain	2015–2017	Single	102	Photon (50 kV)	2.2	No rate reported. Local relapse in one patient
Vaidya/TARGIT-A, 2020 [31]	UK, Europe, US, Australia, Canada	2000–2012	Multi	1,140	Photon (50 kV)	8.6	2.1%
Obi et al., 2020 [32]	Cleveland clinic, US	2011–2019	Single	201	Photon (50 kV)	1.9	2.0%
Ngugen et al., [33]	University of Oklahoma, US	2013–2017	Single	77	Photon (50 kV)	4.6	3.9%
Tallet et al., 2020 [34]	France	2011–2015	Multi	676	Photon (50 kV)	4.5	1.7%
Valente/TARGIT-R, 2021 [35]	US, Canada	2007–2013	Multi	667	Photon (50 kV)	5.1	6.6%
Giap et al., 2022 [36]	University of Florida, US	2010–2017	Single	201	Photon (50 kV)	5.1	2.7%

Table 3 shows the results of two randomized studies comparing IORT-based PBRT versus WBRT. The differences between the low-energy 50 kV photons IORT versus electron-based IORT in terms of IBTR are very divergent. This distinction between low-energy kV results versus electron IORT results had not been recognized in some of the previous papers debating the pros and cons of IORT in breast cancer and needs to be emphasized. The importance of differences in dosimetry between these two modalities as well as the operator dependability of electron IORT usage also needs to be fully understood [37,38].

**Table 3:** Prospective phase III randomized clinical trial results: intraoperative radiotherapy-based accelerated partial breast irradiation versus whole breast irradiation. ELIOT: electron-beam radiotherapy; TARGIT-A: targeted intraoperative radiotherapy during lumpectomy; IORT: intraoperative radiotherapy; WBI: whole breast irradiation; %: percentage

Randomized clinical trial	Number of patients	Inclusion criteria	Follow-up years	Treatment arms	Local recurrence	Toxicity
ELIOT [37] - using electrons	1,305	48–75 years old; <2.5 cm size; cN0	12.4	50 Gy/25 fractions + 10 Gy boost; IORT 21 Gy with electrons	10/15-year IBRT: 1.1%/2.4% WBI vs. 8.1%/12.6% IORT	Reduced skin pigmentation in IORT patients; no difference in fibrosis, retraction, and pain; increased fat necrosis with IORT
TARGIT-A [38] - using 50 kV X-rays	Total: 1,721 IORT vs. 1,730 WBI; pre-pathology: 1,140 IORT vs. 1,158 WBI; post-pathology: 581 IORT vs. 572 WBI	45 years or older; <3.5 cm size; cN0-1	Entire group: 2.4 years; pre-pathology: 8.6 years; post-pathology: 9.0 years	IORT (20 Gy at surface) vs. WBI	Entire group 5 years: 3.3% vs. 1.3%; pre-pathology 5 years: 2.11% vs. 0.95%; post-pathology 5 years: 3.96% vs. 1.05%	Entire group: no differences in wound complications; pre-pathology: no differences in major toxicities; post-pathology: WBI increased grade 3-4 skin toxicities

Table 4 summarizes the use of IORT among special populations and special circumstances [38-42].

**Table 4:** TARGIT-related studies using IORT-based PBRT including IORT alone, IORT used as a boost, IORT used in retrospective settings, IORT in elderly women, and delayed use of IORT. IORT: intraoperative radiotherapy; PBRT: partial breast radiation therapy; EBRT: external-beam radiation therapy; %: percentage

TARGIT-related studies using intraoperative radiotherapy-based accelerated partial breast irradiation
TARGIT-A [38]: The Lancet published the largest international, multicenter, prospective, randomized non-inferiority phase III trial in 2013. Overall mortality was 3.9% for TARGIT-A vs. 5.3% for EBRT. Breast cancer-related deaths were almost the same between the two groups, 2.6% vs. 1.9%, but there were more non-breast cancer deaths with EBRT (1.4% vs. 3.5%) due to deaths from cardiovascular causes and other cancers. After two years and five months of follow-up, the five-year risk of local recurrence was 3.3 % vs. 1.3%
TARGIT-Boost [39]: TARGIT-IORT can boost the tumor bed, followed by EBRT. IORT BOOST was studied by the TARGIT-B trial in 2013 and is still ongoing
TARGIT-Retrospective [40]: TARGIT-R trial is the most extensive retrospective study in North America to analyze the frequency of use, patient selection, and outcomes of IORT. 79% received primary IORT at the time of surgery, 7% had secondary IORT as a delayed procedure, and 14% underwent boost followed by EBRT. After a median follow-up of 23.3 months, local recurrence was 2.3% for all patients treated. The recurrence rate for primary vs. secondary IORT was 2.4% and 6.6%
TARGIT-Elderly [41]: This is a prospective, international, multicenter, single-arm phase II study based on the protocol of TARGIT-A. It recruited 538 elderly low-risk patients (>70 years, cT1 and small cT2, cN0, cM0, and invasive ductal) to confirm the efficacy and toxicity of a single dose of IORT in elderly patients. The expected local relapse rates are 0.5%, 1.0%, and 1.5% after 2.5, 5.0, and 7.5 years, respectively
Delayed TARGIT-IORT [42]: During the TARGIT-IORT trial, another randomized clinical trial was performed simultaneously. In 2004, Vidya launched an additional study comparing delayed TARGIT-IORT with postoperative EBRT in low-risk patients after BCS. It was a non-inferiority, international, multicenter, prospective study. The total number of patients was 1,153. After five years of follow-up, local recurrence was 3.96% vs. 1.05%. However, after median nine-year long-term follow-up, the rates of local recurrence-free survival, mastectomy-free survival, distant disease-free survival, and overall survival were the same

Table 5 summarizes the results from meta-analysis or data mining analysis [42-45]. The data establish the safety and equivalency of outcomes between IORT-based PBRT and WBRT outside of the randomized clinical trial settings. However, the interpretation of the findings presented in Table 5 has to be cautious, given the many shortcomings of meta-analyses and data mining investigations.

**Table 5:** Summary of the SEER data and meta-analysis data on the use of intraoperative radiotherapy in early-stage breast cancer. SEER: Surveillance, Epidemiology, and End Results; EBRT: external-beam radiation therapy; IORT: intraoperative radiotherapy; EBC: early breast cancer; BCS: breast-conserving surgery; RT: radiation therapy; OS: overall survival; DMFS: distant metastasis-free survival; LRFS: local recurrence-free survival

Results of meta-analysis	
1	A meta-analysis by vaidya et al. [42], published in 2015, showed five-year data from randomized trials of partial-breast irradiation vs. EBRT for 1,153 invasive breast cancer patients treated with lumpectomy and found no difference in breast cancer mortality. Partial breast irradiation was better than EBRT for non-breast cancer mortality and total mortality, leading to a 25% relative reduction
2	Juan Lei used the SEER database [43], published in 2019, to investigate the survival outcomes and factors significantly associated with clinical outcomes of IORT compared to whole-breast EBRT for women with EBC. The study enrolled 477,353 patients diagnosed with primary breast cancer between 1998 and 2013. It segregated them into two groups based on whether they received IORT or EBRT after surgery. The results indicate that there is no difference between cancer-specific survival and overall survival
3	Yin Mi used a propensity score matching based on the SEER database [44], published in 2022, to investigate the overall survival and BCS outcomes of IORT compared to no RT in low-risk EBC. The study enrolled approximately 20,000 patients diagnosed with early-stage breast cancer between 2010 and 2019. They separated into two groups based on whether they received IORT and observation after BCS. The results indicate improved BCCS in IORT rather than elimination of RT in low-risk EBC patients
4	A meta-analysis by Lin He in 2021 [45] compared the oncological efficacy of IORT vs. EBRT in EBC patients after BCS. It included 38 eligible studies, with 32,000 analyzed patients. A non-comparative binary meta-analysis calculates the weighted average five-year LRFS, DMFS, and OS. It showed equality in both OS and DMFS but lower LRFS (96.8% vs. 98% in IORT vs. EBRT)

The controversies and debates in the use of intraoperative radiotherapy in breast conservation therapy early-stage breast cancer: statistical aspects of the two randomized studies

TARGIT-A (Accrual From 2000 to 2012) [38]

This was a randomized, non-inferiority trial that compared risk-adapted RT using single-dose targeted IORT (TARGIT) (dose = 20 Gy; n = 1,721) versus EBRT (dose = 50-60 Gy; n = 1,730) for breast cancer patients at 33 centers in 10 countries. The study included the following two groups: those receiving IORT at the time of surgery (pre-pathology) and those receiving IORT as a second procedure (post-pathology). The study reported five-year results for local recurrence, the first analysis of overall survival, and concluded that the pre-pathology IORT timing is ideal.

**Table 6:** Five-year results for local control and overall survival from the TARGIT-A randomized trial. Pre-pathology TARGIT-IORT (n = 1,140), EBRT (n = 1,158); post-pathology TARGIT-IORT (n = 581), EBRT (n = 572). TARGIT-IORT: single-dose-targeted intraoperative radiotherapy; EBRT: external-beam radiotherapy; CI: confidence interval; %: percentage; n: number

Variable	TARGIT-IORT (N = 1,721)	EBRT (N = 1,730)	P-value
Non-breast cancer deaths	1.4% (95% CI = 0.8-2.5)	3.5% (95% CI = 2.3-5.2)	0.008
Breast cancer mortality	2.6% (95% CI = 1.5-4.3)	1.9% (95% CI = 1.1- 3.2)	0.560
Overall mortality	3.9% (95% CI = 2.7-5.8)	5.3% (95% CI = 3.9-7.3)	0.099
Grade 3 or 4 skin complications	4 of 1,720	13 of 1,731	0.029
Five-year local recurrence	3.3% (95% CI = 2.1-5.1)	1.3% (95% CI = 0.7-2.5)	0.042
Pre-pathology cohort local recurence	2.1% (95% CI = 1.1-4.2)	1.1% (95% CI = 0.5-2.5)	0.310
Pre-pathology breast cancer deaths	3.3% (95% CI = 1.9-5.8)	2.7% (95% CI = 1.5-4.6)	0.720
Pre-pathology non-breast cancer deaths	1.3% (95% CI = 0.7-2.8%)	4.4% (95% CI = 2.8-6.9)	0.016
Post-pathology cohort local recurence	5.4% (95% CI = 3.0-9.7)	1.7% (95% CI = 0.6-4.9)	0.069
Post-pathology breast cancer deaths	1.2% (95% CI = 0.4-4.2)	0.5% (95% CI = 0.1-3.5)	0.350
Post-pathology non-breast cancer deaths	1.6% (95% CI = 0.62-3.97)	1.8% (95% CI = 0.7-4.4)	0.320

TARGIT-A Update (Reported in 2020) [31]

This update reported the findings of the TARGIT-A trial, in which 2,298 patients were randomized after their needle biopsy. The post-pathology stratum of 1,153 patients was recruited using a separate randomization table. In the TARGIT-A updated report, with long-term follow-up (median 8.6 years), no statistically significant difference was found between immediate TARGIT-IORT and EBRT outcomes (Table 7). Only the mortality from other causes was significantly lower (45 vs. 74, 95% CI = 0.40-0.86; p = 0.005). This delayed TARGIT study (2004-2013) results are presented in Table 8.

**Table 7:** Ten-year results for local control and overall survival from the TARGIT-A randomized trial. TARGIT-IORT: single-dose-targeted intraoperative radiotherapy; EBRT: external-beam radiotherapy; DCIS: ductal carcinoma in situ; n: number; %: percentage

Variable	TARGIT-IORT (n = 1,140)	EBRT (n = 1,158)	P-value
Non-breast cancer deaths	3.90%	6.30%	0.005
Breast cancer mortality	5.70%	4.90%	0.540
Five-year mortality	3.70%	4.80%	0.130
Five-year local recurrence	2.11%	0.95%	0.280
Five-year local recurrence with or without DCIS	1.30%	0.80%	0.700
Five-year local recurrence with DCIS	0.50%	0.10%	0.740

**Table 8:** Long-term (10-year) results from the TARGIT-A randomized trial in early breast cancer. TARGIT-IORT: single-dose-targeted intraoperative radiotherapy; EBRT: external-beam radiotherapy; n: number; %: percentage

Variable	Delayed TARGIT-IORT (n = 581)	EBRT (n = 572)	P-value
Local recurrence-free survival	83.00%	87.40%	0.052
Invasive local recurrence free survival	83.65%	88.12%	0.051
Mastectomy-free survival	85.89%	86.89%	0.380
Distant disease-free survival	89.33%	89.17%	0.980
Overall survival	90.37%	90.21%	0.800
Breast cancer mortality	3.44%	2.79%	0.500
Mortality from other causes	6.19%	6.99%	0.890

Delayed TARGIT Study (2004-2019) [42]

Four years after accrual began in the TARGIT-A study, patients were randomized after their initial surgery to undergo either EBRT or a further operation to deliver delayed RT to the wound by reopening the original incision. Breast cancer patients who had their cancer excised were randomly enrolled from 28 centers in nine countries. This study’s results crossed the 2.5% margin of non-inferiority; results recommended that immediate TARGIT-IORT should be the preferred treatment over delayed TARGIT-IORT.

Limitations of TARGIT-A Studies

(a) TARGIT A’s five-year preliminary results with a median follow-up of 29 months, with only 35% of the patients having a five-year follow-up at the time of analysis. (b) Unlike the earlier report, long-term results from TARGIT-A published the pre-pathology and the post-pathology stratum separately. (c) In the first publication, with the TARGIT-A approach, the five-year local recurrence rates were inferior to EBRT for both pre-pathology and post-pathology cohorts, although without significant p-values. The IORT was associated with slightly more same-breast recurrences with 3.3% vs. 1.3% in EBRT (p = 0.42), even though this was still within the 2.5% margin for non-inferiority planned at the initial design of the study. (d) The primary endpoint of the TARGIT-A study was local recurrence. Still, for the long-term analysis of the pre- and post-pathology cohorts, a separate statistical analysis plan was drafted with local recurrence-free survival as the primary endpoint. (e) No background risk factors for deaths from non-breast cancer were collected.

Comments on Biostatistics Aspects

(a) The overall statistical strength for TARGIT-A (pre-pathology) is satisfactory and acceptable. (b) Interpreting all three publications was not easy and takes enormous effort. Hence, we created three tables (Tables 6-8) to make it easier for the readers. (c) It is important to understand that pre-pathology results are compelling in favor of IORT. (d) Post-pathology local recurrence rates were higher for the IORT cohort. Hence, the use of this option as a standard practice is not acceptable currently. However, the reasons for differences between pre and post-pathology circumstances need to be analyzed further. This can lead to innovative new ways to overcome these shortcomings in the post-pathology circumstances with appropriately designed future clinical trials. (e) Although the long-term results suggest not using the post-pathology IORT as a standard of practice, we should not shy away from conducting new clinical trials that will have more nuanced selection criteria. The convenience of IORT and overwhelming acceptance by women, thus encouraging them to choose BCT, demand further investigation of this option under post-pathology circumstances utilizing fully informed consent and institutional review board-approved clinical trials scenario with new nuanced selection criteria, including modern next-generation sequencing techniques.

Electron versus 50 kV X-rays dosimetry and the implications on the outcomes

Electron and kV photon beams are often used to deliver IORT treatments. Each has its advantages and disadvantages concerning radiation dose distribution and radiation protection. Commercially available systems based on electron beams are the Mobetron (IntraOp Medical Corporation, Sunnyvale, CA, USA), the Liac, and the Novac IORT systems (Sit Sordina IORT Technologies Spa, Vicenza, Italy). Typical electron energies used for IORT range from 4 MeV to 12 MeV. Spherical applicator sizes range from 3 cm to 10 cm in diameter, and elongated applicators are available up to 8 × 20 cm^2^. The advantage of electron IORT is that tumors can be adequately treated from a depth of 0 to about 4 cm with 90% of the prescribed dose. The ratio of the surface dose to the maximum dose is about 0.9. Therefore, any tumor cells located 0-4 cm away from the applicator surface can adequately be treated. Large tumor beds can also be treated with larger applicators and applicators of different dimensions. One major potential issue with electron IORT from a dosimetric perspective is that the electron beam is directed mainly in a forward direction. Therefore, the surgeon must gather the target tissue and place it within the field of the electron applicator. The treatment challenges are like a clinical setup for external-beam electron therapy where the lateral margin and depth must be estimated without three-dimensional imaging. Therefore, lateral coverage of the tumor bed may not always be guaranteed, especially because a margin of 1-2 cm is usually needed to ensure coverage dosimetrically beyond just the target border. This can be challenging in the treatment of the tumor bed after a BCS procedure as the primary target is largely the lumpectomy cavity in every direction (360°). The main dosimetric advantages of the electrons are their much higher penetrating ability in depth and their capability to treat a much larger flat area than kV X-rays.

In the case of IORT for the tumor bed cavities created after lumpectomy, the following facts have to be considered to ascertain the shortcomings of electron beam IORT in BCT: (a) the depth of microscopic/subclinical disease in the tumor bed cavities in need of IORT doses in BCT after lumpectomy is only a few millimeters, so the depth dose advantages of the electron beam-based IORT are irrelevant. (b) Also, the whole cavity in a 360° dimension is at risk; with the forward-directional electron beam dose profile, electron beam IORT has major disadvantages compared to the low-kV IORT.

The two-commercial kV IORT systems are the Zeiss Intrabeam System (Carl Zeiss Meditec AG, Jena, Germany) and the Xoft system (Xoft Inc., Sunnyvale, CA, USA). Both systems use 50 kV X-rays. Treatments are delivered using specifically designed flat, surface, and spherical applicators. The significant advantage of kV IORT is that many applicators are commercially available and can be inserted directly into the lumpectomy cavity for the breast. The entire lumpectomy cavity is treated with little possibility of geometrical miss except for the unknown depth of any possible microscopic extension if the surgical margin is not sufficient enough. The incision can also be made to be smaller for an excellent cosmetic outcome for the patient. Another advantage is that because we are dealing with kV photon beams, the radiation protection issue is less of a problem [46]. The main disadvantage of the kV photon beam systems is the steep dose gradient so that the ratio of the dose at the surface of the applicator to the dose at a depth of, say, 1.0 cm and 2.0 cm is about 3.5 and 6, respectively. Therefore, for a prescription dose of 20 Gy at the surface, the dose at 1 cm from the surface is about 6 Gy and reduced to 3 Gy at 2 cm, limiting the treatment depth.

Several major randomized trials of IORT versus conventional EBRT listed above used IORT treatment units of earlier versions. The ELIOT trial involved using the Liac and Novac systems, specifically electron modalities [47]. The TARGIT-A trial featured the Intrabeam system from Zeiss, a kV modality. Notably, the difference in local control outcomes between these two trials is striking though not directly compared. Both were statistically inferior regarding local control to the external beam. Still, the TARGIT-A demonstrated a roughly 5% reduction in control, whereas the ELIOT trial showed a quite significant 15% loss in control rate [31]. Without a direct head-to-head comparison, it may be difficult to say definitively what the issue is. Still, we postulate that despite the penetration advantages of the electron units, the geometric proximity of the kV units to the target area may indeed lead to a significant clinical benefit in reducing the risk of geometric miss in breast cancer lumpectomy cavities. Though the reasons for the difference may not be entirely known, it is our opinion that these differences should not be ignored when discussing treatment options with patients. The two modalities should not necessarily be considered equivalent in the quality of treatment delivery. However, the authors would like to note that in other disease sites, such as the abdomen and pelvis, the above dosimetric considerations may not apply.

Cost-benefit considerations

The International Atomic Energy Agency (IAEA) has a nearly complete (90%) list of RT equipment, linear accelerators and teletherapy (cobalt) machines [48]. IAEA’s Directory of Radiotherapy Centres (DIRAC) has a database of 7,600 RT centers with about 13,000 external therapy units and 2,600 brachytherapy units. DIRAC has developed a metric called RT utilization (RTU), defined as the proportion of cancer patients being treated with at least one course of RT during the entire natural history of cancer. This is about 50-55% in developed countries such as the USA. IAEA predicts that the RTU should be higher (about 70-80%) in less developed countries because the cancers are more advanced at presentation due to a lack of cancer screening programs, resulting in a higher need for RT. However, the real RTU in developing countries is closer to 25-40% due to insufficient RT units and the cost of RT equipment [48].

In a recent paper by Healy et al. based on IAEA data, the cost of cobalt machines versus linear accelerators in terms of not only the initial purchase cost but also the overall maintenance cost over its useful lifetime were compared and are shown in Table 9 [49].

**Table 9:** Indicative costs of cobalt-60 machines and linear accelerators (linacs) in Euro. MLC: multileaf collimator; EPID: electronic portal imaging device; RVS: record and verify system This table is reproduced from Healy et al. [49], and permission was obtained from the licensed content publisher Elsevier.

Details	Cobalt-60 machine excluding MLC, EPID and RVS	6 MV linac with MLC, EPID and RVS	Multi-energy linac (including electrons) with MLC, EPID, and RVS
Up-front cost including 1 year of warranty	600,000	900,000	1,500,000
14-year service contract	500,000	1,260,000	2,100,000
Source exchanges (2 of)	280,000	N/A	N/A
Total cost over 15 years	1,380,000	2,160,000	3,600,000

The cost of cobalt-60 machines was lower than that of linear accelerators. Table 10 compares the pros and cons of the two modalities. The choice of the type of machine needs to be determined not only on the initial cost and/or overall lifetime cost but also on the availability of engineering staff who can maintain the machines, medical physics/dosimetry staff who can help provide high-quality and safe treatments, and high rate of (over 98%) upkeep time for the machines. The worst cost is to buy an expensive machine that goes unused [49].

**Table 10:** Characteristics of cobalt-60 machines and linear accelerators (linacs). FFF: flattening-filter-free; N/A: not applicable This table is reproduced from Healy et al. [49], and permission was obtained from the licensed content publisher Elsevier.

Item	Cobalt- 60 machine	Single-energy linac	Multiple-energy linac
Physical characteristics	Relatively simple	Complex electric equipment	More complex electrical equipment
Dosimetry	Limited by source size, 1 MeV gamma ray beam energy	Smaller source size, versatile dose rate, 6 MV X-ray beam energy	Smaller source size, versatile dose rate including FFF, multiple X-ray beam energies up to 20 MV
Shielding requirements	Concrete bunker and maze	Concrete bunker and maze with thicker walls	Concrete bunker and maze with thicker walls and possibly neutron door
Staff	Baseline	Higher than baseline particularly in medical physics, engineering and IT support. More training required	Higher than single energy linac, particularly in medical physics. More training required
Costs	Baseline	Slightly higher than baseline	Significantly higher than baseline
Security	Security measures required for source in place for source exchange	Easily compliant	Easily compliant
Source exchange	Every 5 years	N/A	N/A
Clinical use	Can easily provide simple treatment techniques	Can more easily provide complex treatment techniques	Can more easily provide complex treatment techniques, allows superficial treatments with electrons
Patient throughput	Affected by source decay	Can be compromised by poor maintenance	Can be compromised by poor maintenance

The above facts lead us to conclude the use of IORT use in breast cancer. (a) Breast cancer patients require a specialized surgical center to get lumpectomies and/or mastectomies. (b) Travel to such centers for rural societies/less developed geographies is inevitable. (c) The cost and maintenance of an IORT machine are lower/easier than a teletherapy/linear accelerator. (d) The hospital stays of a maximum of 5-10 days (for lumpectomy plus IORT) is more acceptable and practical for patients and families when they travel a sizeable distance from their home than stay for surgery followed by weeks of EBRT. (e) Sophisticated techniques such as using stereotactic radiotherapy as an alternative to IORT can be done in developed economies; however, it is cost-prohibitive in less developed economies.

The above discussion shows that even in resource-lean situations, IORT can have a role that can improve the cost-benefit and cost-efficiency ratios in addition to practical implementation and acceptability rates. The above logic is further supported by a health economics analysis comparing IORT versus EBRT by Vaidya et al. [50]. They concluded that the IORT approach has a higher cost-effective gain and quality-adjusted life advantages than EBRT. These analyses were for a developed nation (United Kingdom); the advantages will likely be different for less developed economies or resource-limited settings, as most patients are usually diagnosed at a later stage making them ineligible to be treated using IORT on its own. On the other hand, IORT can be used as a boost dose to the whole breast irradiation; breast EBRT can be hypofractionated plus an IORT boost to shorten the whole course of RT. However, these new concepts need to be tested under clinical trial circumstances. In the United States, the cost of an IORT machine (either electron IORT or low-energy X-ray machine) is about one-sixth to one-tenth of a linear accelerator capable of modern EBRT for breast (depending on the sophistication of the machine with capabilities such as active breath control, intensity-modulated RT, volumetric modulated arc therapy, daily on treatment imaging (cone-beam CT/X-ray volume imaging), and adaptive MRI-based RT. The cost ratio of a simple linear accelerator likely to be used in less economically advanced geographies versus a simple IORT machine is unlikely to be any different. The above considerations are important for policymakers when investing in *women’s cancer programs* in any geography, economically advanced or less advanced. The authors of this report are not in a position to recommend the choice of modality because each circumstance requires detailed strategic planning.

The importance of patient and family-focused care, patient’s choice, risk determinations by patients, and informed consent

**Figure 1:**
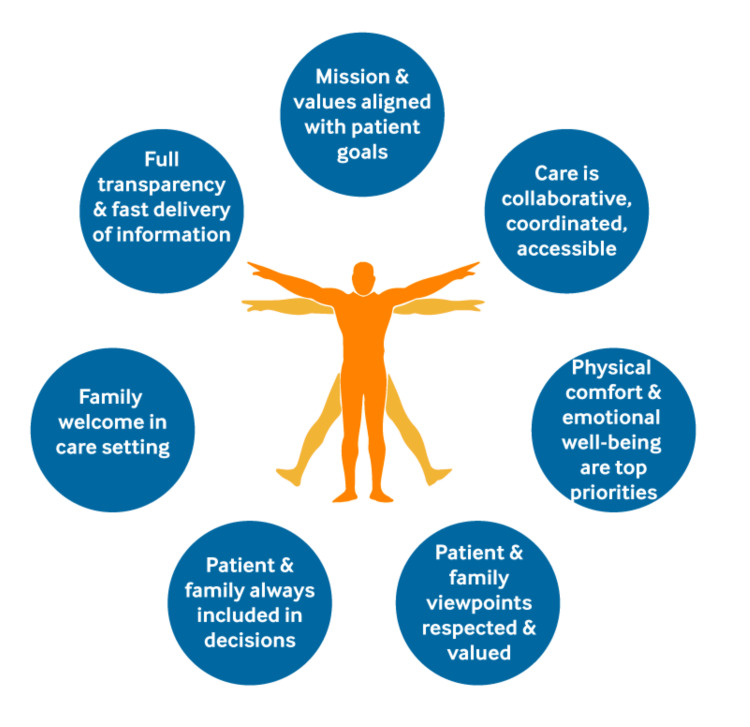
Schematic representation of patient-centered care. This image is reproduced from NEJM Catalyst [51], and permission was obtained from the licensed content publisher Massachusetts Medical Society.

Patient and family-centered care (PFCC) is defined as one that encourages active collaboration and shared decision-making between patients, families, and providers to design and manage a customized and comprehensive care plan [51]. The tenets of PFCC are outlined in Table 11 and Figure 1 below. In a review of patient-centered care approaches in healthcare, Constand et al. concluded three necessities [52]. These include communications, partnerships, and health promotion. These three aspects should be among the top priorities of the caregiving healthcare team and the team’s discussions with patients and families.

**Table 11:** Patient-centered care: what we need to know? This table is adapted from NEJM Catalyst [51], and permission was obtained from the licensed content publisher Massachusetts Medical Society.

The elements of patient-centered care	
1	The healthcare system’s mission, vision, values, leadership, and quality improvement drivers are aligned with patient-centered goals
2	Care is collaborative, coordinated, and accessible. The right care is provided at the right time and in the right place
3	Care focuses on physical comfort as well as emotional well-being
4	Patient and family preferences, values, cultural traditions, and socioeconomic conditions are respected
5	Patients and their families are an expected part of the care team and play a role in decisions at the patient and system levels
6	The presence of family members in the care setting is encouraged and facilitated
7	Information is shared fully and in a timely manner so that patients and their family members can make informed decisions

As so many choices exist in managing EBC with comparable outcomes, PFCC is crucial in helping patients and families reach optimal decisions for the given circumstances [13]. In this respect, the care team must provide the facts and guide the patient and family to make the appropriate decision for her/him/them and help achieve it. This is even more important in the use of IORT in EBC because so many considerations (Table 12) go into decision-making for the patient and family.

**Table 12:** Women with early breast cancer: options, pros, and cons. RT: radiation therapy

In favor of mastectomy		Against mastectomy	
1	Probably the shortest time in terms of getting all treatments done	1	The most disfiguring
2	A psychological feeling that the cancer is gone and no chances of it coming back	2	A sense of loss of self-esteem
3	Least interruption in taking care of family and occupational demands	3	If reconstruction surgery is added, the benefits of least interruption listed under advantages of mastectomy are lost
		4	Potential loss of sexual satisfaction
In favor of external beam radiotherapy (six weeks) or hypofractionated shorter 1-3-week course		Against external beam radiotherapy ( six weeks) or hypofractionated shorter 1-3-week course	
1	Breast preservation	1	Weeks of life interruption
2	No loss of self-esteem	2	Potential side effects of RT
3	Advantages in sexual function results	3	Cardiac and lung doses, albeit small doses with modern RT techniques
4	Long-term Class I evidence of equivalent or even improved survival (meta-analysis data) compared to mastectomy		
In favor of intraoperative radiotherapy		Against intraoperative radiotherapy	
1	Breast preservation	1	The uncertainty of needing further weeks of RT if the final pathology indicates the need for such RT
2	No need for daily visits for RT	2	The shortest duration of follow-up in the clinical trials
3	The shortest time commitment possible	3	Some controversial discussions in the peer review literature, often confusing to even healthcare providers, let alone patients/families
4	The least interruption in life and work	4	Too many guidelines from professional societies, although with only minimal variations in recommendations
5	Potentially the least side effects for RT		

Given all of the above, PFCC is even more critical in the decision-making in EBC, and fully informed consent, spending ample time with patients and families, and not rushing them to make rapid decisions cannot be overemphasized.

Professional society guidelines and consensus recommendations: finding a *middle way*

Guidelines and consensus statements (GCS) from expert specialty societies are necessary and handy tools to help clinicians, patients, and families make decisions in situations where black-and-white recommendations are often impossible. GCS often evolves and changes and is fine-tuned as new evidence emerges. That is the current situation in using IORT as a one-time alternative for week(s) of external-beam PBRT in EBC. The fact that multiple GCS exist makes it clear that a cookie-cutter approach will not be helpful. Extreme statements for and against confuse patients, families, and non-specialized practicing physicians.

GCS from major professional societies representing different specialties, such as breast surgeons, radiation oncologists, and brachy therapists, are summarized in Table 13, which is more for PBRT than specifically for IORT.

**Table 13:** Guidelines and consensus statements from expert specialty societies on partial breast radiation therapy (IORT being a type of PBRT). ABS: American Brachytherapy Society; ASTRO: American Society of Radiation Oncology; ASBrS: American Society of Breast Surgeons; DCIS: ductal carcinoma in situ; PBRT: partial breast radiation therapy; IORT: intraoperative radiation therapy

Criterion	ABS update	ASTRO update	ASBrS update
Age	≥45 years	≥50 years; 40–49 years if all other criteria met	≥45 years for all tumor types
Histology	All invasive subtypes; DCIS	All invasive subtypes; pure DCIS	All invasive subtypes; DCIS
Tumor size	≤3 cm	≤3 cm	≤3 cm
T stage	Tis, T1, T2	Tis, T1, T2	Tis, T1, T2
Margins	No tumor on ink for invasive, ≥2 mm for DCIS	Close margins ok	No tumor on ink for invasive tumors or tumors involved with DCIS; ≥2 mm for DCIS

Table 13 reveals more commonalities than differences in general terms of PBRT; however, when it comes to using IORT, there are more controversies [53-55]. To find a *middle way*, the following strategies are recommended for clinicians and patients/families, as outlined in Table 14 and Table 15.

**Table 14:** Finding a middle way: strategies for clinicians in recommending IORT in early breast cancer. IORT: intraoperative radiation therapy Recommendations for clinicians by the authors of this paper in finding a middle way.

Recommendations for clinicians	
1	Take a multidisciplinary team approach in decision-making that includes breast surgeons, radiation oncologists, pathologists, breast radiologists, medical oncologists, medical geneticists, and other cancer care professionals
2	Take an individualized, patient and family-focused approach; assess the patient/family’s risk/benefit comfort level and compare the notes with your colleagues.
3	Take more than one session of discussion with the patient/family; avoid information overload in one session
4	Educational materials can be beneficial
5	Conduct the discussions in a non-examination room setting
6	Avoid treating patients: with multifocal/multicentric tumors; triple-negative tumors; node-positive tumors; tumors too close to the skin; BRCA-positive patients, to name a few
7	Patients should be treated in a clinical trial setting at this time, at least a registry study; however, in individual patients who are uncomfortable participating in clinical studies, the consensus tumor board discussions and decisions can be very helpful
8	Second opinions, even within one’s practice, can be used effectively

**Table 15:** Finding a middle way: strategies for patients and families. Recommendations for patients and families by the authors of this paper in finding a middle way.

Recommendations for patients and families	
1	Take time to decide; most early-stage breast cancers, especially hormone receptor-positive tumors, do not spread rapidly in one or two weeks, so taking a week to 10 days to make a decision is acceptable
2	Get a second opinion if that would help
3	Consult your family; however, the patient’s choice is final and needs to be supported by the rest of the family
4	The research information is constantly evolving; although there may be some increase in the recurrence within the treated breast compared to external-beam radiotherapy, this depends on many factors: how closely do your cancer care doctors work as a team?; is there a tumor board where they discuss the details of each patient and develop professional consensus decisions?; what is the volume of patients with similar conditions they treat?; is the cancer care center an academic center that treats many types of cancers?; are the cancer care team members willing to take time to explain, be transparent, and not rushed?; how involved is the medical physics team with the treatment planning?; does the cancer care center have permanent medical physicists on site?; how extensive are the quality assurance efforts in the cancer care center one uses?
5	The willingness to discuss with other patients who have received the same treatment can be beneficial

Cancer care providers must be cognizant of the truth that GCS is not written in stone. They are supposed to guide professionals to, in turn, guide patients and families to make the right decision for the right patient at the right time, given individual patient/family’s circumstances (financial, job-related, child care, elderly care, employer’s support, transportation issues, cancer care support services in that geographic area, etc.), and comfort level with a specific decision and, sometimes, with particular care provider/care providing team. The most important aspect is that all cancer care providers and team members should ultimately respect the decision of patients/families and help them reach their goals.

A patient/family-focused, nuanced approach to BCT without a bias of the *one-size-fits-all* approach of the GCS will help women in states such as Mississippi and other rural and resource-lean locations. Some women might be willing to take a 1-3% additional risk of breast recurrence if they could have BCS and RT done intraoperatively to improve convenience. This may give women more confidence in breast preservation and encourage a higher percentage of women to undergo routine breast screening and choose BCS. Not all institutions have an IORT unit. In these situations, other novel RT techniques are emerging and can be offered to patients with EBC. For patients who meet the criteria for a partial breast radiation approach, stereotactic partial breast irradiation with emerging ultra-hypofractionation schedules are being investigated, including single fractions [56,57]. These options may be desirable to patients who do not have access to an IORT machine, have transportation difficulty, live in rural areas, work full-time, or have other responsibilities that require minimal time away from these regular routines.

**Figure 2:**
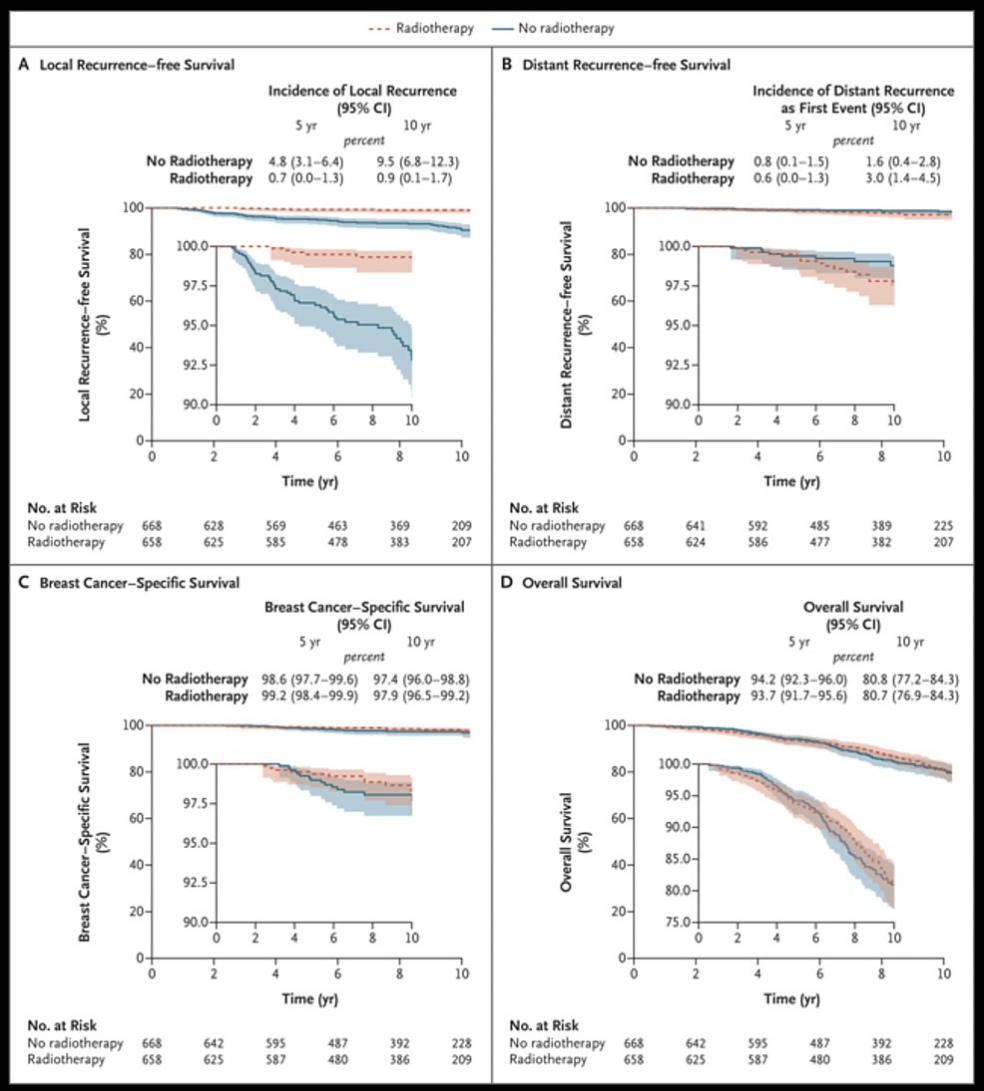
The endpoints of local recurrence, distant recurrence, breast cancer-specific survival, and overall survival rates with or without radiation therapy. yr: year; CI: confidence interval; %: percentage This image is reproduced from Kunkler et al. [58], and permission was obtained from the licensed content publisher Massachusetts Medical Society.

One of the recent options can be even completely omitting RT in EBC in very selected populations [58]. In a recent study, Kunkler et al. reported the results of a phase III randomized study in women 65 years or older with eligibility criteria similar to that of IORT eligibility for EBC. In total, 1,326 patients were studied and managed with WBRT or observation. Overall survivals were equal; however, there were very high local recurrence rates in the observation arm, as shown in Figure 2 (9.5% versus 0.9% between observation vs. RT arms, respectively) [58].

This again speaks more in favor of IORT in low-risk patients considering their QOL (e.g., the fear of recurrence as well as the inconvenience of requiring surveillance periodically) and the added costs of surveillance. As precision medicine concepts progress further, we will be able to identify who among women with EBC will not need even IORT [59,60]. A PFCC cannot be overemphasized when helping our patients make these decisions [13]. Further discussions on the role of precision medicine, radiobiotherapy, and genomic medicine in breast cancer RT are beyond the scope of this review. However, we can observe that the support for precision medicine-based clinical trials in radiotherapy in EBC in improving survival and local control outcomes, QOL, and patient/family satisfaction in care is the need of the day [61].

## Conclusions

This review and perspective can serve many purposes. Foremost, it should clarify many misunderstandings of the data of the clinical trials, both for and against IORT in breast cancer. The dosimetric aspect of the report and the biostatistical clarifications can be helpful to bring some closure to the controversies and help in consensus development. It is important to realize that every patient with an even identical clinical, pathological, genetic, imaging, and biomarker profile may not still have the same socioeconomic status/educational background nor the same comfort level with the so-called GCS-dictated ideal treatment. It is still the cancer care provider’s responsibility to bow to the wishes of the patient/family to facilitate that choice. At the same time, the practitioner also relays optimal survival and balances QOL outcomes.

It is hoped that this report will stimulate more clinical, epidemiological, and patient/family participation in cancer care studies, especially in breast cancer. The potential halo effects of breast preservation potential and the convenience of IORT may positively impact the willingness of women to come forward for breast cancer screening promptly and according to the required timelines. This especially needs to be investigated among RSEDPs by our population science colleagues. What can be learned from such studies in breast cancer can then be applied to many other cancers in the RSEDPs.

And, finally, this communication will hopefully lead to some educational materials for women with EBC and their families. Again, this can be used to help patients and practitioners make decisions more easily on a subject with so many facts and so many differences in opinion (often passionate) in a way that is reader-friendly and non-scientific.
